# Aplastic Anaemia with Microfilaria in Marrow Aspirate

**DOI:** 10.4084/MJHID.2012.019

**Published:** 2012-03-16

**Authors:** Narender Tejwani, Seema Tyagi, Jasmita Dass

**Affiliations:** Department of Hematology, All India Institute of Medical Sciences, Ansari Nagar, New Delhi, India

**Dear Editor,**

Filariasis is a parasitic disorder and is endemic in many parts of the world especially in the tropical countries including India.^1^ The disease presents itself predominantly in lymphatic and cutaneous forms and caused by *Wuchereria bancrofti* and *Brugia malayi.*^2^ The adult worms can be demonstrated in a variety of aspiration cytology smears. Previously rare associations have also been reported in special stain done for acute leukemia, staging marrow for Hodgkin’s lymphoma and suspected lymphoma patients.^3^ However, there are limited numbers of reports describing the presence of this parasite in bone marrow aspirate smears 4 and even rare is the presence of this disease along with aplastic anemia.^5^ There is a single case reported by Hemachandran M et al in 2003^5^ where aplastic anemia developed in a patient with coexisting varicella and Wuchereria bancrofti infection. Sharma S et al in 2006^6^ reported 6 cases of *Wuchereria bancrofti* in bone marrow aspirate smears with the interesting finding of marrow hypoplasia in five of these cases. We report here a patient presenting with features of marrow failure and incidentally found to have microfilaria of *Wuchereria bancrofti.* The case is being reported due to this rare association.

A 9 year old female presented to hematology outpatient department with chief complaints of generalised weakness and high grade off & on fever for last 20 days. On examination she had pallor, fever, epistaxis and bleeding from gums. There was no lymphadenopathy or sternal tenderness. On systemic examination there was no organomegaly. She was found to have pancytopenia. Hemoglobin was 2.8 gm/dl, total leukocyte count was 3000/μl and platelet count was 17000/μl. A presumptive diagnosis of bone marrow failure was made. Bone marrow aspirate and biopsy was advised for confirmation.

Jenner giemsa stained bone marrow aspirate smears showed the presence of few paucicellular fat rich fragments in a diluted smear (Figure 1). The myelogram showed predominantly small mature lymphocytes (93%) and plasma cells (5%). Also seen on screening were few microfilariae of *Wuchereria bancrofti* (Figure 2). The bone marrow biopsy showed a hypocellular marrow with predominance of lymphocytes and plasma cells (Figure 2, inset). Overall cellularity was reduced to 10%.

The index case presented with complaints of fever and weakness which are one of the most common presentation in aplastic anemia. These patients are susceptible to many infections due to reduced immunity. The diagnosis of parasitic infections may not be suspected clinically. Treatment for parasitic infection is not a part of routine treatment for fever in these patients. Treatment of fever is usually done by broad spectrum antibiotics which are not going to be effective for the treatment of parasitic infections. The fever due to parasitic infection may thus lead to unnecessary usage of antibiotics. The presence of fever may lead to further drop in blood counts. Therefore diagnosis of parasitic infection like microfilaria is required for specific therapy. To conclude it is important to keep a high suspicion of parasitic infections in these patients with aplastic anemia and the marrow aspirate slides should always be screened for the presence of these parasites.

## Figures and Tables

**Figure 1:**
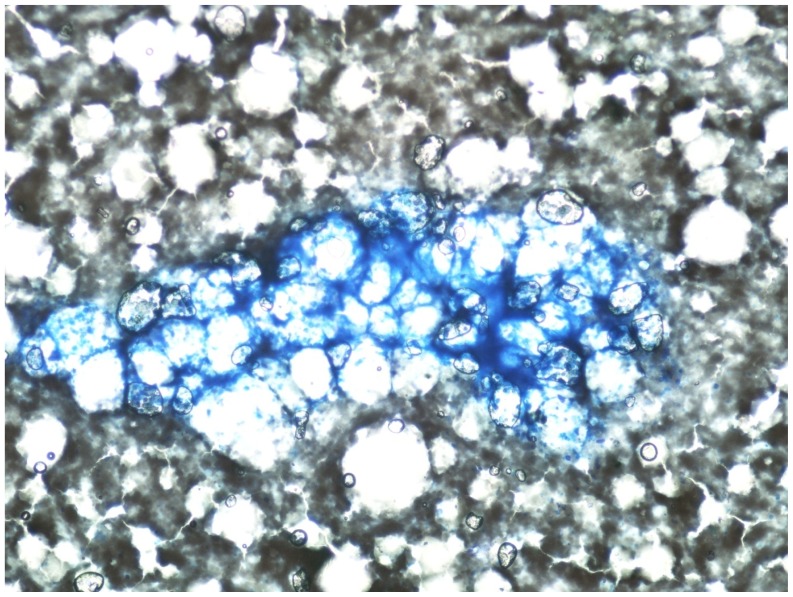
Bone marrow asiprate showing fat rich fragment (giemsa stain, 400 X)

**Figure 2:**
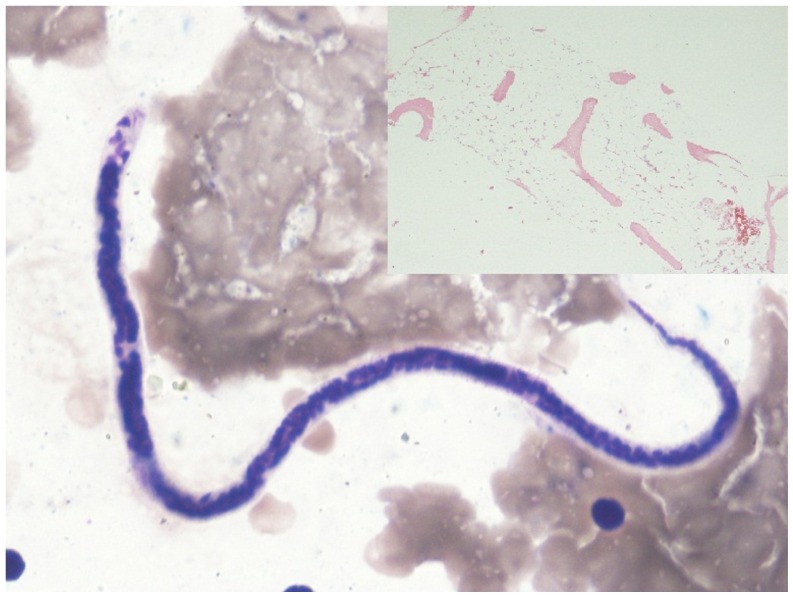
Wuchereria bancrofti in marrow aspirate (giemsa stain, 1000X) with hypoplastic marrow ( Biopsy, inset, H&E stain, 100X)
